# The capacity of wastewater treatment plants drives bacterial community structure and its assembly

**DOI:** 10.1038/s41598-019-50952-0

**Published:** 2019-10-15

**Authors:** Young Kyung Kim, Keunje Yoo, Min Sung Kim, Il Han, Minjoo Lee, Bo Ram Kang, Tae Kwon Lee, Joonhong Park

**Affiliations:** 1 School of Civil and Environmental Engineering, Yonsei University, Seoul, Republic of Korea; 2 Department of Environmental Engineering, Yonsei University, Wonju, Republic of Korea; 3 Present Address: Department of Environmental Engineering, Korea Maritime and Ocean University, Busan, Republic of Korea

**Keywords:** Ecology, Microbiology, Ecology

## Abstract

Bacterial communities in wastewater treatment plants (WWTPs) affect plant functionality through their role in the removal of pollutants from wastewater. Bacterial communities vary extensively based on plant operating conditions and influent characteristics. The capacity of WWTPs can also affect the bacterial community via variations in the organic or nutrient composition of the influent. Despite the importance considering capacity, the characteristics that control bacterial community assembly are largely unknown. In this study, we discovered that bacterial communities in WWTPs in Korea and Vietnam, which differ remarkably in capacity, exhibit unique structures and interactions that are governed mainly by the capacity of WWTPs. Bacterial communities were analysed using 16S rRNA gene sequencing and exhibited clear differences between the two regions, with these differences being most pronounced in activated sludge. We found that capacity contributed the most to bacterial interactions and community structure, whereas other factors had less impact. Co-occurrence network analysis showed that microorganisms from high-capacity WWTPs are more interrelated than those from low-capacity WWTPs, which corresponds to the tighter clustering of bacterial communities in Korea. These results will contribute to the understanding of bacterial community assembly in activated sludge processing.

## Introduction

Wastewater treatment encompasses the processes that convert contaminated water into a sufficiently clean state that can be discharged to surface water with minimal adverse environmental impact. Wastewater treatment plants (WWTPs) employ a series of processes, namely, preliminary treatment, primary settling, secondary treatment, secondary clarification, tertiary treatment, disinfection, and sludge processing, each of which is designed to remove different types of pollutants. Of these processes, secondary and tertiary treatments are where most organic matter, nutrients, and other pollutants are removed, and these are commonly achieved using biological methods^1,2^. Thus, bacterial community composition, diversity, and dynamics, which are shaped by both operating conditions and influent characteristics^3,4^, are the major factors that determine the performance of a wide range of biological treatments and activated sludge (AS) processing^5–8^. Many studies have analyzed the responses of the key microorganisms for wastewater treatment under different WWTP operation practices^2,9^.

Capacity is a complex function of physical constraints (e.g. reactor volumes and equipment capacities), influent characteristics (e.g. microbial composition and organic type) and operation factors (e.g. sludge retention time [SRT] and hydraulic retention times [HRT]) that are strongly associated with sludge processes including sludge flocculation, process stability, and microbial flexibility^10,11^. WWTP capacities around the world are inherently variable. In Seoul, which has a population of 10 million, four extremely large-scale wastewater treatment facilities have been operated. Actually, the city is known to have one of the largest wastewater treatment plants in the world. Because the wastewater used by the large number of citizens flows into WWTP through the pipeline, the influent can contain variety of microbes as well as organic compounds. Since the microorganisms entering bioreactor in WWTPs are mainly from the influent, microorganisms in the influent containing various substrates may influence community compositions or population diversity in the bioreactor^12^. Meanwhile, some of newly developing Asian cities such as Ho Chi Minh or Hanoi started to manage relatively small-scale wastewater treatment facilities in small administrative districts or large buildings due to geographical limitations and construction/installation costs^13^. The biochemical composition of wastewater generated in such a small area is relatively homogeneous, and bacterial communities with a similar function are likely to be selected for biological reactors. The remarkable different capacities of the Seoul’ and Vietnamese WWTPs allow us to explore an interesting question, i.e., if bacterial composition in a bioreactor is influenced only by WWTP capacity or by other geographical factors.

The effects of capacity on bacterial communities in WWTPs were indirectly evaluated by comparison of 16S rRNA gene sequencing in previous studies. WWTPs in Hong Kong, which have a large WWTP capacity (>200,000 tonnes/day), showed significantly higher bacterial diversity compared to WWTPs in Singapore and the United States, which have relatively small in the capacity (8000-55,000 tonnes/day)^6^. Although Proteobacteria and Bacteroidetes at the phylum level dominant in all samples, the dominant genera showed geographical characteristics. Global studies on the diversity of bacterial communities in 269 WWTPs (23 countries on 6 continents) also observed that their geographical turnover is scale-dependent and driven by stochastic processes (dispersal and drift)^14^. WWTPs with larger capacity, the greater the diversity of microorganisms introduced, may increase the probability of stochastic processes occurring in community assemblies^12^. Despite recent advances in our understanding of the effects of capacity on the bacterial diversity and structure of WWTPs, information on the bacterial interactions or community assembly remains elusive. This information is essential for identifying the key players in the biological treatment process and understanding the sludge assembly for stable wastewater treatment.

We collected AS samples from a total of eight WWTPs in Korea and Vietnam, which have similar operation parameters and treatment processes but different the capacities (flow rates). Deep sequencing of 16S rRNA gene and co-occurrence network analysis were performed to understand the effects of WWTP capacity on bacterial diversity and community assembly. We also evaluated the key players (hub members) to interpret their functional potentials in WWTPs with different capacities.

## Materials and Methods

### Sampling

Influent and AS from eight different WWTPs were used for this study. A total of 49 samples (8 influents and 41 AS) were collected from high-capacity WWTPs in Seoul, South Korea, between November 2014 and April 2015 (collectively referred to as ‘KR_WT_’). AS samples were obtained from anaerobic, anoxic and aerobic zones, respectively, in each WWTP and pooled into three or four AS samples to reduce potential sampling bias. The designed capacities and operating temperatures of these WWTPs ranged between 500,000 and 1,000,000 tonnes/day, and 10 °C and 16 °C, respectively. A total of 18 samples (7 influents and 11 AS) were collected from low-capacity WWTPs in Hanoi and Ho Chi Minh, Vietnam, between January and February 2015 (collectively referred to as ‘VN_WT_’). The designed capacities and operating temperatures of these WWTPs ranged between 1000 and 10,000 tons/day and 24 °C and 28 °C, respectively. The monthly average values for five water quality indicators [biological oxygen demand (BOD), chemical oxygen demand (COD), suspended solid (SS), total nitrogen (TN) and total phosphorus (TP)] of influent and effluent, were measured using environmental standard methods^15^. To take representative samples, sampling was performed at a minimum of 20 to 30 cm below the water surface. All samples were stored at −80 °C before extracting DNA.

### DNA extraction and sequencing

Total genomic DNA from each AS was extracted using a FastDNA SPIN Kit for Soil (MP Biomedicals, Santa Ana, CA, USA) according to the manufacturer’s protocol. DNA quality was checked using an ND-1000 spectrophotometer (NanoDrop Technologies, Wilmington, DE, USA), and samples were stored at −20 °C.

A 16S amplicon polymerase chain reaction (PCR) forward and reverse primer targeting the V3 to V4 regions of the 16S rRNA gene^16^ was used to amplify the 16S rRNA gene from the extracted DNA. Amplification was conducted using a C1000TM thermal cycler (Bio-Rad, Hercules, CA, USA) with the following conditions: 94 °C for 1 min, 30 cycles at 94 °C for 1 min, 50 °C for 1 min, and 72 °C for 2 min with a final extension at 72 °C for 5 min. PCR products were purified by gel electrophoresis bands isolated using a QIAquick PCR Purification Kit (Qiagen, Hilden, Germany). The purified amplicons were sequenced by Macrogen Inc. (Seoul, Korea) using an Illumina Miseq sequencer. The data sets were deposited at the National Center for Biotechnology Information Sequence Read Archive database under accession number PRJNA533065.

### Community analysis

Sequences were processed using MOTHUR software version 1.39.5^17^ according to the standard operating procedure (http://www.mothur.org/wiki/MiSeq_SOP)^18,19^ with minor modifications^20^. Briefly, sequences longer than 466 bp were discarded, and those that remained were screened with start and end positions of 10,364 and 25,316, respectively, after being aligned to the SILVA database Release 132^21^. Taxonomy was classified using RDP 16S rRNA as a reference trainset 16^22^. The VSEARCH in MOTHUR was used to filter chimeras. Operational taxonomic units (OTUs) were clustered using the OptiClust algorithm with a 97% sequence similarity cutoff ^23^.

### Network analysis

Co-occurrence networks were inferred based on a Spearman correlation matrix^24^ and constructed using only significant correlation^25^. The cutoff for correlation coefficients was determined to be 0.6 and the cutoff for p-values was 0.001^26^. The cutoff was chosen by variance of interaction strength. All network construction steps were computed in R using code adapted from https://github.com/ryanjw/co-occurrence ^27^.

### Statistical analysis

Statistical analysis was implemented using the R platform. The significance of the differences in bacterial diversity (richness and evenness) between WWTP types was evaluated by comparing Wilcoxon rank-sum test p-values. Non-metric multidimensional scaling (NMDS) was performed using the ‘vegan’ package based on the relative abundance profiles at OTU levels. Water quality parameters were used for loading vectors contributing the ordination by permutation test (p-value < 0.05). Permutational multivariate analysis of variance (PERMANOVA) was performed using the ‘vegan’ package, and the permuted p-value was obtained by 10,000 times of permutations.

## Results and Discussion

### Characteristics of WWTPs in Korea and Vietnam

Table 1 summarises the operating parameters and removal efficiencies for each of the eight WWTPs in Korea and Vietnam. Most of biological treatments of WWTPs in this study have been operated by anaerobic/anoxic/oxic (A2O) or modified Ludzack-Ettinger (MLE) processes for treating domestic wastewater, except for one WWTP that used sequencing batch reactor (SBR) for industry wastewater. AS VN_WT_ in this study was constructed by Korean construction companies, similar processes as KR_WT_ were applied. Regardless of country and region, WWTPs had similar overall operating parameters and removal efficiencies. Flow rates and SRT were significantly different between countries (p-value < 0.05) whereas other parameters (HRT, mixed liquor SS [MLSS] and pH) were comparable to each other. The differences in WWTPs in the two countries can be explained by historical and social reasons. When Seoul was developed in earnest in the 1970s, residential plans and WWTP construction plans were established spontaneously. The pipe construction and WWTP expansion plans could be considered at the same time; thus, the size of KR_WT_ was successfully expanded as to handle approximately 1,000,000 m^3^/day due to the explosive increase in the population of Seoul. However, Vietnam had many buildings already built in the city, so it was difficult to build large WWTPs due to geographical limitations and construction/installation costs^13^. VN_WT_ has recently to build on a small scale because WWTPs have been built in a large new building or a small town. SRT is closely related to the flow rate because it is affected by the amount of sludge generated by the capacity of the WWTPs and the required MLSS concentrations. Taken together, the flow rates of the two regional WWTPs are considered to have the largest difference in operating parameters.

**Table 1:** Major operating parameters of WWTPs in this study. Asterisk indicates significant differences between KR_WT_ and VN_WT_.

WWTPs	KR1	KR2	KR3	KR4	VN1	VN2	VN3	VN4	p-value
Wastewater source	Domestic	Domestic	Domestic	Domestic	Domestic	Domestic	Domestic	Industry	—
Treatment process	MLE/A2O	MLE	MLE/A2O	MLE	MLE	MLE	A2O	SBR	—
_avg_SRT (days)	10	9	10	9	16	16	16	18	0.0247*
_avg_HRT (hours)	10	7	7	6	9	9	8	6	0.6704
_avg_Flow rate (m^3^/day)	850,000	1,500,000	1,700,000	900,000	1,000	1,300	1,000	18,000	0.0294*
_avg_MLSS (mg/L)	2,700	2,800	2,500	2,800	3,200	2,700	2,800	3,000	0.1379
pH	6.9	6.9	7.0	6.9	7.1	6.9	6.8	7.0	0.8770
COD_rem_ (%)	95.6	92.9	94.8	92.1	93.9	92.7	92.5	92.4	0.3123
BOD_rem_ (%)	92.6	91.7	94.3	92.9	92.5	93.4	91.8	93.6	0.9439
TN_rem_ (%)	91.8	96.8	90.4	93.9	97.7	92.7	95.5	96.3	0.2312
TP_rem_ (%)	97.1	97.8	96.5	92.3	99.8	99.0	92.9	97.9	0.4851

MLE, Modified Ludzack-Ettinger; SBR, Sequencing Batch Reactor; A2O, Anaerobic/Anoxic/Oxic process.

The water quality parameters of influents and effluents were monitored to compare treatment performances for KR_WT_ and VN_WT_ samples (Supplementary Fig. S1). The influent composition exhibited no significant difference between the two countries, except that KR_WT_ had a wider distribution in BOD and COD and a narrower distribution in SS and TN. The wider distribution of BOD and COD suggested that there are a various types of substrates entering wastewater or a conversion of organic matter during travel of wastewater from the source to WWTP^28^. Such a trend was absent in VN_WT_ samples because the contaminants produced in the same facilities or small town were always similar and similar types of substrates flowed into the influent. These results indicated that WWTPs with large capacity are likely to have various COD and BOD. The high fluctuation in influent TN and SS could be the unique characteristic of small WWTPs, as individuals have greater contribution to the composition of wastewater. Thus, changes in water usage patterns of a small portion of inhabitants can result in large change in influent characteristics. For instance, about 30% to 40% of household wastewater is from bathrooms^29^ and because small WWTPs serve fewer households compared to the large WWTPs, minor changes in the pattern of individuals occupying bathrooms at any given hour can have a large effect on the influent quality of small WWTPs. The high COD of VN_WT_ samples suggests different design criteria of WWTPs and/or AS less climatised for degrading recalcitrant organic substances which requires a series of complex microbial actions^30^.

### Bacterial diversity and composition

**Figure 1:**
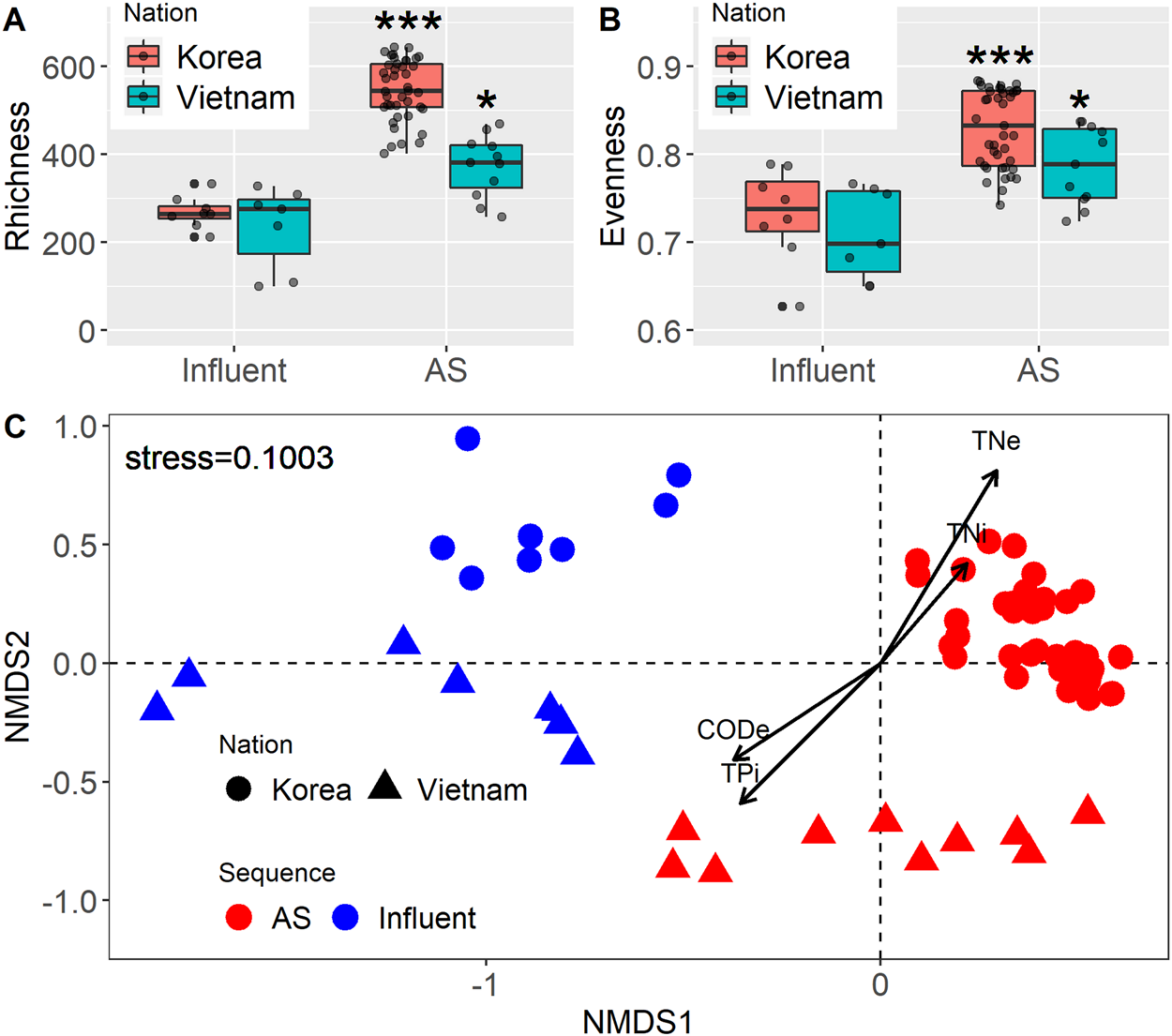
Comparison of microbial diversity and community structure between WWTPs in Korean and Vietnam. (**A**) Richness (number of observed OTUs), **(B)** evenness, **(C)** NMDS plot showing microbial communities present in this study (k = 2; stress = 0.1003). Water quality parameters indicated by arrows are significant parameters (p-value < 0.05) contributing to each ordination. CODe, COD in effluent; TNe, TN in effluent; TNi, TN in influent; TPi, TP in influent.

Sequencing obtained 27,581 ± 10,031 reads and 28,915 ± 10,535 reads of quality reads from influent and AS of KR_WT_ and VN_WT_, respectively. Good’s coverages were all above 95%, indicating that the number of sequences was enough for sampling the communities. The richness (observed OTUs) of KR_WT_ influent exhibited less variable compared to that of VN_WT_ influent (Fig. 1A). Such a large spread could be due to the fact that minor change in water usage can have a large effect on influent characteristics for small WWTPs, as discussed in Section 3.1. The richness increased in AS for both WWTP types with comparable variances. Nevertheless, a larger increase was observed for KR_WT_ AS (p-value < 0.001; average richness = 546), whereas the increase for VN_WT_ AS was less (p-value < 0.05; average richness = 373). The evenness of KR_WT_ and VN_WT_ exhibited similar trends to the richness (Fig. 1B). It is widely recognised that parameters such as pH, total organic carbon, essential nutrients, and dissolved oxygen (DO) have a significant impact on bacterial communities in various ecosystems^31,32^. Thus, bacterial diversity and evenness may reflect not only the WWTP design but also influent characteristics, which are strongly affected by the cultural, social and environmental differences in each region^32^. Gao *et al*.^33^ reported that COD and pH are the primary variables of influent that can affect the bacterial community of AS. Although pH data were unavailable in this study, COD was one of the parameters that exhibited a large variation in KR_WT_ influent. The large variability of COD can be linked to the influx of various types of substrates in the influent, which might be manifested by increasing the diversity of microorganisms depending on the substrate specificity.

The differential distribution of bacterial populations between KR_WT_ and VN_WT_ on an NMDS plot was statistically confirmed using PERMANOVA (R = 0.786, p-value < 0.05; Fig. 1C). In general, the y-axis separated the sequences based on their origin, with KR_WT_ on the positive side and VN_WT_ on the negative side of the plot, whereas the x-axis separated sequences based on the wastewater treatment phase, with more negative values for the influent than the AS. The water quality parameters including (TNe: TN in effluent, TNi: TN in influent, CODe: COD in effluent, TPi: TP in influent) were selected as significant loading vectors (p-value < 0.05) contributing the ordination in the NMDS plots. In previous studies, several WWTPs processing influents with differing characteristics and using different operating parameters differed significantly in their bacterial community structures^6,32,34,35^. However, in this study, the bacterial communities found at the same wastewater treatment phase were more related than those from the same location. This is reasonable because although KR_WT_ and VN_WT_ influents have different characteristics, and undergo different biochemical reactions in the sewer network, this does not change the fact that they are generated from human activities. Furthermore, two of VN_WT_ were from WWTPs designed for in-complex wastewater treatment. Thus, as the influent was always composed of similar types or components of pollutants due to the limited source of influent in the in-complex WWTP, the composition of bacterial communities might be specifically formed according to each WWTP in Vietnam. Likewise, the similar bacterial communities of AS also contributed to the clustering of the samples from different locations. Although the designed capacities differed at each WWTP, their main purpose is the same – that is, the removal of organic matter and excess nutrients. Thus, although the designs for WWTPs of KR_WT_ and VN_WT_ samples may be different, both types were built based on well-established biological methods that rely strongly on the development of specific bacterial communities for their performance. Notwithstanding the numerous factors that could contribute to the differences in AS bacterial communities, such as treatment capacity, operation parameters, and local environmental conditions, the shift was insufficient to break the clustering trend.

**Figure 2:**
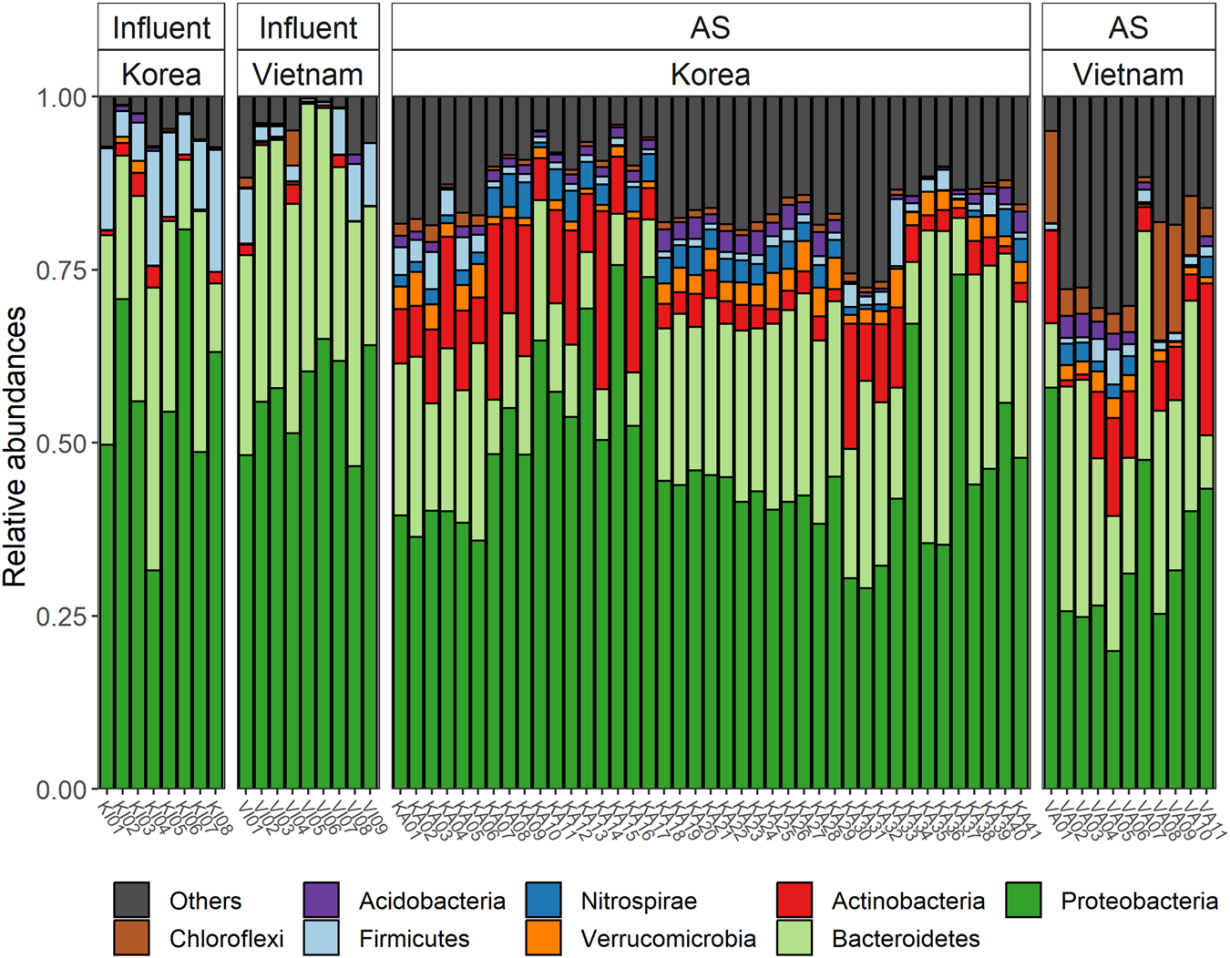
Phylum level microbial community composition in influents and WWTPs.

In this study, Proteobacteria was the most abundant phylum in all samples of both KR_WT_ and VN_WT_, accounting for 40% to 60% of the total effective bacterial sequences (Fig. 2). This is consistent with other results of bacterial communities in influent^36^ and AS^6,37^. The other substantial phyla identified in this study included Bacteroidetes (20–30%), Firmicutes (5–10%), and Actinobacteria (3–8%). Proteobacteria are believed to be involved in the removal of organic pollutants, such as nitrogen, phosphorus, and aromatic compounds^5,37^. Wagne and Loy^5^ surveyed eight different WWTPs in which Beta-, Alpha-, and Gammaproteobacteria, as well as Bacteroidetes, were frequently found, in that order, although the relative ratios were somewhat variable. Previous studies on AS using microarrays^8^ or cloning^1^ similarly found that the same four groups identified in this study were dominant (around 80–85%) in bacterial communities, followed by a few other major (average abundance >1%) phyla, including Chloroflexi, Nitrospirae, Acidobacteria, Candidatus Saccharibacteria, and Verrucomicrobia. A few phyla in this study, such as Bacteroidetes, Firmicutes, Actinobacteria, Nitrospirae, Candidatus Saccharibacteri, and Verrucomicrobia, differed significantly between Korean and Vietnamese AS samples (p-value < 0.05).

These results suggest that bacterial communities develop differently depending on the capacity of WWTP, which is consistent with previous studies^3,4^. Wastewater engineers have tried to develop treatment processes by creating specific conditions for harnessing particular microorganisms^6,38^. Specific environmental conditions, including abiotic factors (e.g. temperature, DO, pH, organic matter, HRT and SRT) and interspecies interactions (e.g. competition, symbiosis and predation), may influence on the selection of specific bacterial populations from the influent wastewater community that are better suited to the bioreactor. Then, specific niches to the capacity of WWTP are created with low dispersal rate, and significant different communities could be assembled in the bioreactors. Previous studies suggested that the geographical distribution of WWTPs might be an important and influential factor in the variation of WWTP bacterial communities^32,34,35^.

### Network analysis

**Figure 3:**
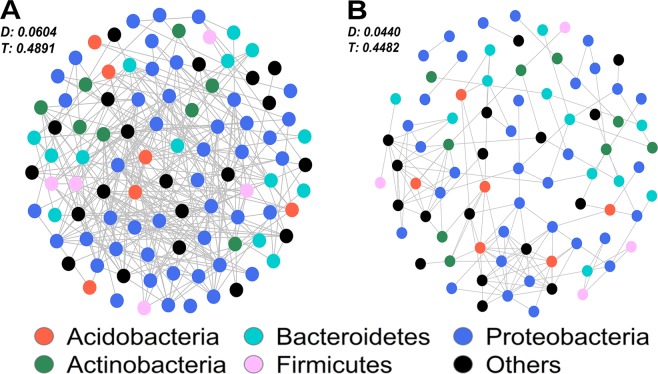
Network of co-occurring microbial OTUs of AS based on correlation analysis for **(A)** Korea (nodes = 98, edges = 287) and **(B)** Vietnam (nodes = 82, edge = 146). A connection stands for a strong (Spearman’s rho > 0.6) and significant (p-value < 0.001) correlation. Nodes are colored according to phylum. D, Density; T, Transitivity.

Diversified functional traits are closely associated with ecological niche differentiation, and network topology allows us to focus on the patterns of bacterial relationships that develop within a given ecosystem^39^. Network analysis was incorporated to explore the assembly of bacterial communities in the high-capacity KR_WT_ and low-capacity VN_WT_ (Fig. 3). KR_WT_ samples had more nodes (98) and edges (287) as well as higher network density (D = 0.0604) and transitivity (T = 0.4891) compared to VN_WT_ samples (nodes = 82, edges = 146, D = 0.0440, and T = 0.4482). These results indicated that microorganisms from high-capacity WWTPs are more interrelated than those from low-capacity WWTPs, which correspond to the tighter clustering of KR_WT_ in NMDS (Fig. 1). These results indicated cooperation among diverse microorganisms in AS for wastewater treatment. Furthermore, this interaction was broader and richer in large-capacity WWTPs, which could be foreseen from the significantly higher diversity and evenness of KR_WT_ compared to that of VN_WT_ (Fig. 1). These results suggested that higher wastewater treatment capacity promotes bacterial diversity and interaction in AS. High-capacity WWTPs serving larger populations receive more diverse organic matter and nutrients from more sources than the low-capacity WWTPs. This facilitates not only broader metabolic reactions that utilise diverse substrates found in wastewater but also the expansion of ecological niches responsible for bioconversion^40^. This competition or mutualistic interaction might contribute to stimulating taxonomic and functional diversity^41^. Frequent physicochemical and nutritional variation in a single ecosystem strongly influences bacterial community structure, but co-occurrence network patterns are quite persistent and interactive because of ecological rearrangements, in such a way that an important fraction of OTUs come to fill the interactive roles occupied by other OTUs in the network when facing strong environmental changes^42^.

**Figure 4:**
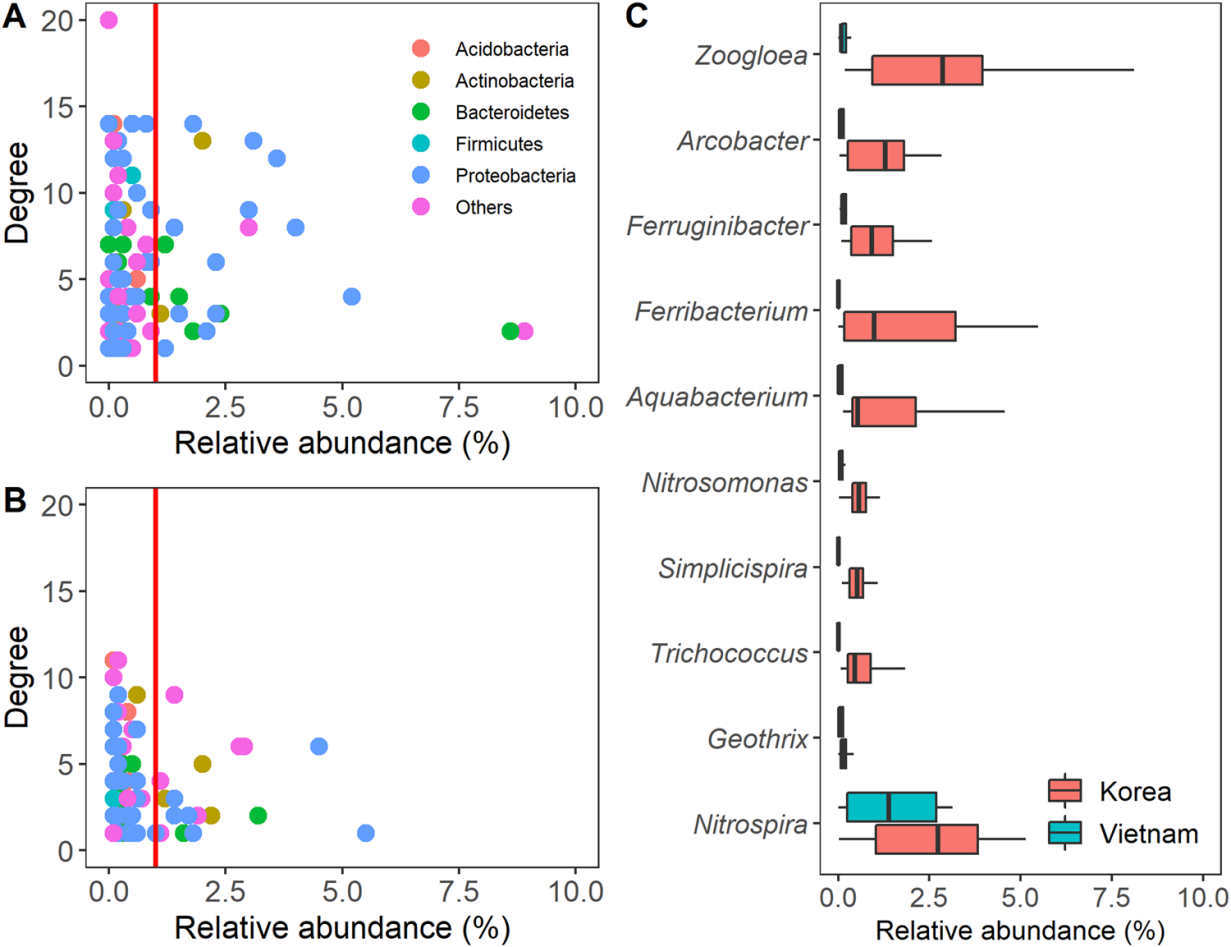
Relationship between the number of degree and relative abundance of each node (OTU) in AS for Korea **(A)** and Vietnam **(B)**. The dominant phyla members are highlighted. **(C)** Relative abundances of the members with high degree in this study.

Hub members were defined as microbes with a high degree (number of connections) of co-occurrence (top 5%) and those are key players in sustaining the overall community^43^. More hubs were observed in KR_WT_ (10 hubs) than VN_WT_ (6 hubs), but most of the detected hubs were of relatively low abundance (<1% of total), particularly those in VN_WT_, which were close to zero (Fig. 4A,B). It is worth noting that KR_WT_ had a significantly higher number of hub-like members (>3 degrees) with abundances greater than 1%, but VN_WT_ members with abundances of more than 1% were very rare. Meanwhile, hubs in VN_WT_ were assigned to others (non-dominant phyla in this study) or Acidobacteria, whereas those in KR_WT_ were mainly affiliated to diverse phyla, including Acidobacteria, Actinobacteria, Firmicutes, and Proteobacteria. These results indicate that diverse microorganisms in KR_WT_ tended to be metabolically active and established heterogeneous interactions with other microorganisms that aided wastewater treatment. Bacterial diversity has been shown to be a major factor associated with network complexity, suggesting induced niche differentiation, functional redundancy, and process stability^44^. These may result in the maintenance of performance recovery, operational stability, and bacterial flexibility in KR_WT_ being more resilient to common disturbances in influent characteristics^11^.

Most members with high degree had a significantly higher relative abundance in Korea compared to Vietnam (p-value < 0.05), except for *Nitrospira* and Geothrix (Fig. 4C). Hub members have been shown to significantly correlate with the function and performance of wastewater treatment^37^; thus, the members could serve as biological indicators for each region. Hub members were commonly found in other WWTPs, as described in previous studies^34,35^. For instance, *Zoogloea* and *Trichococcus* are the main agents for the flocculation of AS because they form extracellular gelatinous matrices^34,45^, whereas *Ferruginibacter* and *Arcobacter* are capable of hydrolysing some organic matter^46^. *Nitrosomonas* plays a core role in nitrification in WWTPs^47^. Interactions between microorganisms and members with high degree may perform key biogeochemical processes (e.g., organic removal, nutrient mineralisation, nitrification and phosphorus removal) and stabilise sludge flocs. Such information can provide a basis for targeted manipulation of AS community for the stable and efficient operation of the WWTPs. If AS can be manipulated, the efficiency of wastewater treatment can be increased with reduced treatment time, and the function of sludge can recover quickly when the sludge is damaged by external disturbances.

## Conclusion

This study provides evidence of the impact of capacity on bacterial diversity and community assembly in WWTPs. Although the capacity itself is not directly related to bacterial communities and relationships, changes in COD, SRT and other conditions depending on capacity, could indirectly influence bacterial diversity and its assembly. We have shown that high-capacity WWTPs contain relatively high bacterial diversity and that their bacterial communities are more interrelated than those of low-capacity WWTPs, which results to a tighter clustering of KR_WT_ in NMDS. These differences seem to be induced by the probability of stochastic processes in the AS assembly in different capacities of WWTPs. Co-occurrence network analysis provides information on key players in AS, which may be the basis for manipulating the AS stability in biological treatment processes. The findings support the hypothesis that capacity is a key factor influencing AS bacterial community and its assembly, providing a fundamental bioreactor engineering implication to optimize AS depending on capacity of WWTPs.

## Supplementary information

Supplementary information
