# Supplementary material for: The capacity of wastewater treatment plants drives bacterial community structure and its assembly
Source: Sci Rep. 2019 Oct 15;9:14809. doi: 10.1038/s41598-019-50952-0 (PMC6794251; doi:10.1038/s41598-019-50952-0)
Supplement: Supplementary file 1 — Supplementary information [file 41598_2019_50952_MOESM1_ESM.docx]

**The capacity of wastewater treatment plants drives bacterial community structure and its assembly**

**Young Kyung Kim^1,†^, Keunje Yoo^1,3,†^, Min Sung Kim^2^, IL Han^2^, Minjoo Lee^1^, Bo Ram Kang^2^, , Tae Kwon Lee^2,*^, Joonhong Park^1,*^**

^1^School of Civil and Environmental Engineering, Yonsei University, Seoul, Republic of Korea

^2^Department of Environmental Engineering, Yonsei University, Wonju, Republic of Korea

^3^Current Address: Department of Environmental Engineering, Korea Maritime and Ocean University, Busan, Republic of Korea

^†^Both authors contributed equally to this manuscript.

***Correspondence**:

Tae Kwon Lee; Joonhong Park

tklee@yonsei.ac.kr; parkj@yonsei.ac.kr

Supplementary Material

# Supplementary figures


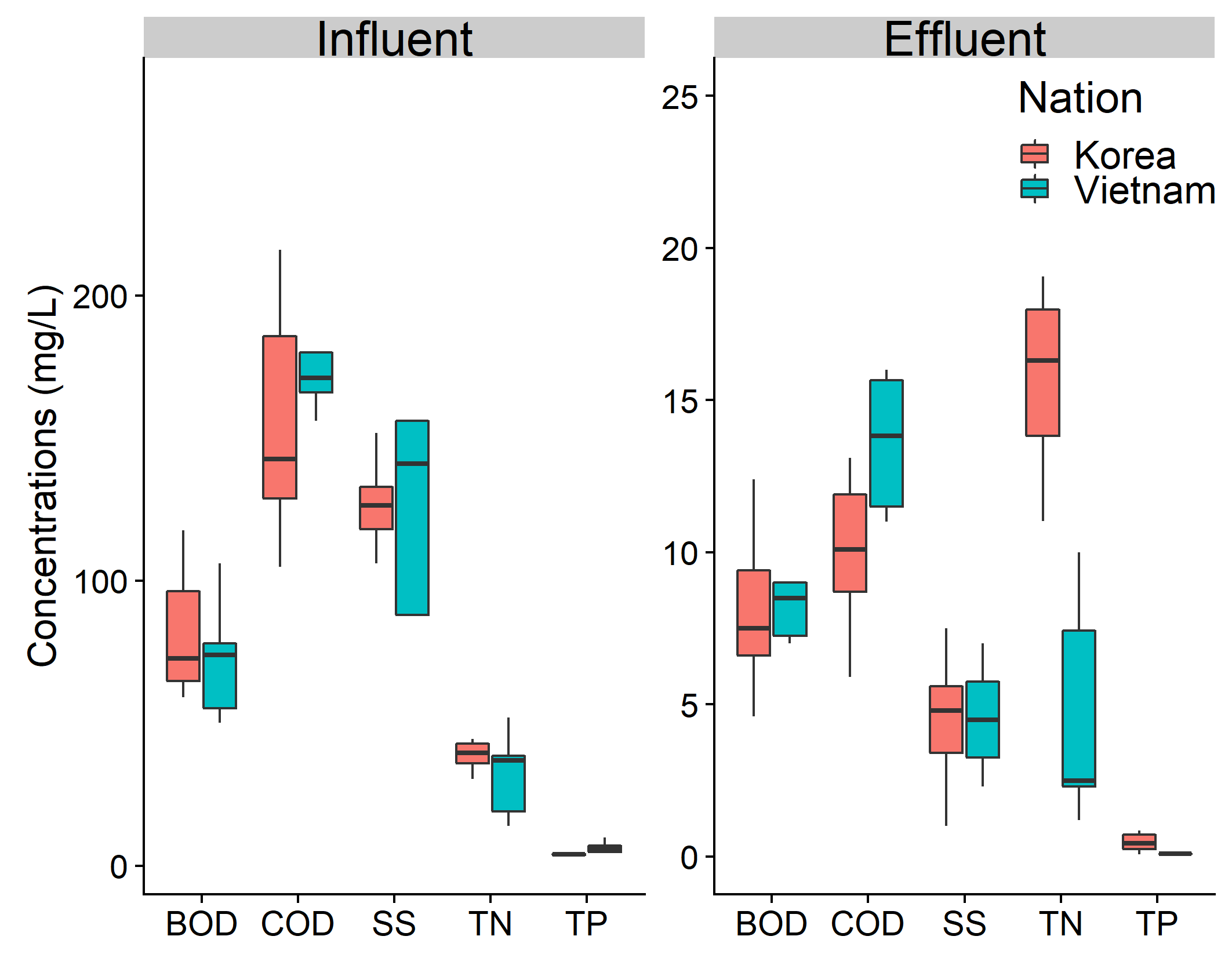


**Supplementary Fig. S1.** Water quality parameters for the influent **(A)** and effluent **(B)** in WWTPs in Korea and Vietnam.
